# A Meta-Analysis of Growth Differentiation Factor-15 and Prognosis in Chronic Heart Failure

**DOI:** 10.3389/fcvm.2021.630818

**Published:** 2021-11-05

**Authors:** Jin-Wen Luo, Wen-Hui Duan, Lei Song, Yan-Qiao Yu, Da-Zhuo Shi

**Affiliations:** ^1^Beijing University of Chinese Medicine, Beijing, China; ^2^National Clinical Research Center for Chinese Medicine Cardiology, Xiyuan Hospital, China Academy of Chinese Medical Science, Beijing, China

**Keywords:** growth differentiation factor-15, chronic heart failure, prognosis, meta- analysis, systematic review

## Abstract

**Background:** Previous studies had reported increased circulating concentrations of growth differentiation factor-15 (GDF-15) in chronic heart failure (CHF), suggesting the potential prognostic significance of GDF-15 in this setting. To verify the relationship between the circulating GDF-15 levels and prognosis of CHF patients, we conducted an updated evidence-based meta-analysis.

**Methods:** A comprehensive literature retrieval of PubMed, EMBASE, and Cochrane library was performed to collect the qualified studies that analyzed the prognostic value of GDF-15 in CHF from the inception of these online databases to September 25, 2021. The hazard ratio (HR) calculated for logGDF-15 of all-cause death and the related 95% confidence interval (CI) in multivariate analysis were used to measure the effect size. Additionally, subgroup analyses stratified by characteristics of the study participants were conducted for incremental evidence of GDF-15 in CHF with different clinical status.

**Results:** A total of ten eligible studies involving 6,244 CHF patients were finally taken into the quantitative analysis. Results in the random-effects model indicated that there was an increased risk of 6% in all-cause mortality with a per 1LnU increase in baseline GDF-15 concentration (HR: 1.06, 95% CI: 1.03–1.10, *P* < 0.001). In stratified analyses, the association of GDF-15 with risk of all-cause mortality was found among chronic ischemic HF patients (HR:1.75, 95%CI: 1.24–2.48, *P* = 0.002), while the association was not found among chronic nonischemic HF patients (HR:1.01, 95%CI: 1.00–1.02, *P* = 0.219).

**Conclusion:** The elevated GDF-15 is associated with an increased risk of all-cause mortality in CHF, especially, among CHF patients with ischemic etiology. The circulating GDF-15 might be a prognostic indicator in CHF patients.

**Registration Number:** https://www.crd.york.ac.uk/PROSPERO; CRD42020210796.

## Introduction

Heart failure (HF), the terminal stage of multiple cardiovascular diseases (CVD), is a complex clinical syndrome with high morbidity and mortality (1). In spite of guideline-directed management, the prognosis of chronic heart failure (CHF) remains poor and the mortality exceeds 50% in five years (2). It is therefore crucial to identify accurate and sensitive biomarkers predicting the outcome of CHF. B-type natriuretic peptide (BNP) and N-terminal pro B-type natriuretic peptide (NT-pro BNP) mainly synthesized by ventricular cardiomyocytes related with cardiac overload, have been established as two effective biomarkers for diagnosis and predicting prognosis of HF (2, 3). However, the expression of BNP and NT-proBNP are affected by other factors, such as atrial fibrillation, renal function, age, and obesity, which lead to a confounder of these biomarkers in clinical practice. Additionally, HF is a systemic clinical syndrome rather than a single pathological process, in which inflammation and oxidative stress play an important role (4). Accordingly, the circulating biomarkers in other pathophysiologic pathways may provide a supplementary information for the risk stratification of HF.

Growth differentiation factor-15 (GDF-15), a stress-responsive cytokine, is a member of the transforming growth factor-β (TGF-β) cytokine superfamily. It is expressed in response to several pathologic processes including inflammatory reaction, oxidative stress and myocardial ischemia (5). Basic data have previously shown the remarkably increased expression of GDF-15 in ventricular or atrial cardiomyocytes under pathological conditions associated with myocardial ischemia as well as mechanical stress and demonstrated the cardioprotective and antihypertrophic effects of GDF-15 in mice (6–8). Clinical studies also observed a higher circulating concentration of GDF-15 in patients with CHF compared with healthy individuals (9–11). Moreover, certain studies in patients with CHF revealed that GDF-15 level was positively correlated and the disease severity, indicating that GDF-15 might act as a novel prognostic biomarker of CHF in risk stratification and prediction of adverse outcomes (12–14).

However, there are conflicting data regarding the association between GDF-15 with the risk of adverse outcomes in CHF patients (15–17). Previous meta-analysis on the association of GDF-15 with HF outcomes did not assess the value of circulating GDF-15 in the prognostic prediction in patients with stable CHF population (18). We therefore performed an updated meta-analysis to determine that whether high-level GDF-15 is linked with adverse outcomes of CHF.

## Methods

### Search Strategy and Selection Criteria

This meta-analysis was reported in accordance with the PRISMA (Preferred Reporting Items for Systematic Reviews and Meta-Analyses) guidelines (19).

At the beginning of this meta-analysis, two independent reviewers (JW Luo and YQ Yu) conducted a systematic, computer-based literature search of PubMed, EMBASE, and Cochrane Library from the inception of the databases to October 1, 2020. On September 25, 2021, we conducted a second literature retrieval to supplement related studies. The following Medical Subject Headings were used to identify relevant studies: “growth differentiation factor 15”, “GDF-15”, “heart failure”, “cardiac failure”, and “cardiac dysfunction”. A manual search of references for related researches was also performed. The literature retrieval was restricted to human studies and papers written in English. A detailed searching strategy is presented in Supplementary file.

The inclusion criteria were: (1) prospective or retrospective follow-up studies which examined the association between GDF-15 and risk of CHF outcomes; (2) Patients diagnosed with CHF with either reduced or preserved left ventricular ejection fraction (LVEF), without age, sexual, and racial limitations; (3) Available data of hazard ratio (HR) and the 95% confidence interval (CI) for all-cause mortality in multivariate analysis. Studies with enrollment of end-stage HF patients were excluded, since up to 75% of patients with end-stage HF had a life expectancy of less than 1 year (20). End-stage HF was defined as HF with severe symptoms and/or signs at rest, recurrent hospitalizations despite guideline-directed management and therapy, requiring heart transplant and mechanical circulatory support (21). Studies rated as low quality were not included either. Low quality studies were defined as literatures with a score of less than 7 in the Newcastle-Ottawa-Scale (22).

Investigators then reviewed the obtained 957 records to identify the eligible studies according to a standard protocol. Where disagreements occurred, discussions were done among the researchers or a third investigator (WH Duan), a specialist in the field, was consulted.

### Quality Assessment and Data Extraction

The Cochrane risk-of-bias assessment tool was not employed due to the study type of non-randomized trials. The Newcastle-Ottawa-Scale (NOS), designed for case-control study and cohort study, was used to assess the methodological quality of each selected study. The NOS is mainly comprised of three dimensions: selection of participants, comparability among groups, and outcome assessment, ranging from 0 to 9 stars. Articles with seven stars and above were rated as high-level quality.

The following data were extracted from each included study: first author's name, publication year, study design, location, number of participants, characteristics of patients at baseline (age, sex, preexisting conditions, and BMI), follow-up period, HR, and 95% CI for all-cause mortality in multivariate-adjusted model. In instances where there was insufficient information, we contacted the corresponding author.

### Statistical Analysis

This study used HR and 95% CI as a measure of the predictive value of GDF-15 in CHF. In each included study, the HR for logGDF-15 was calculated. Therefore, the recorded HR represented the risk of a per 1LnU increase in baseline circulating concentration of GDF-15. Both Cochran's Q test and *I*^2^ index were used to measure the heterogeneity among all studies (23). The random-effects model was adopted for the summary of effect size in instances where significant heterogeneity (*I*^2^ > 50%, *P* < 0.05) existed, while the fixed-effects model was applied in all other instances.

In addition, we performed subgroup analyses stratified by clinical status (NYHA class or LVEF), ischemic etiology (percentage of subjects with ischemic disease < 60% or ≥60%), baseline concentration of GDF-15 (< 2000ng/L or ≥2000ng/L), age (< 65 or ≥65), study design (cohort study or *post-hoc* analyses of randomized clinical trials [RCTs]), year of publication (≤ 2015 or > 2015), sample size (≤ 500 or >500) and follow-up duration (≤ 36 or > 36 months).

Sensitivity analysis was then conducted to estimate the weight of every study in global assessment. Moreover, meta-regression analyses were employed for the identification of potential variables that contributed to the pooled estimate of prognostic value of GDF-15. Finally, Begg's funnel plot (24) with logHRs and the corresponding standard errors (SEs), and Egger's test (25) with z statistics, were used to examine the underlying publication bias. Utilization of the “trim-and-fill” method (26) enabled us to estimate the number of unpublished studies. All the statistical analyses were done in STATA software version 12.

## Results

### Characteristics of Selected Studies and Quality Assessment

**Figure 1:**
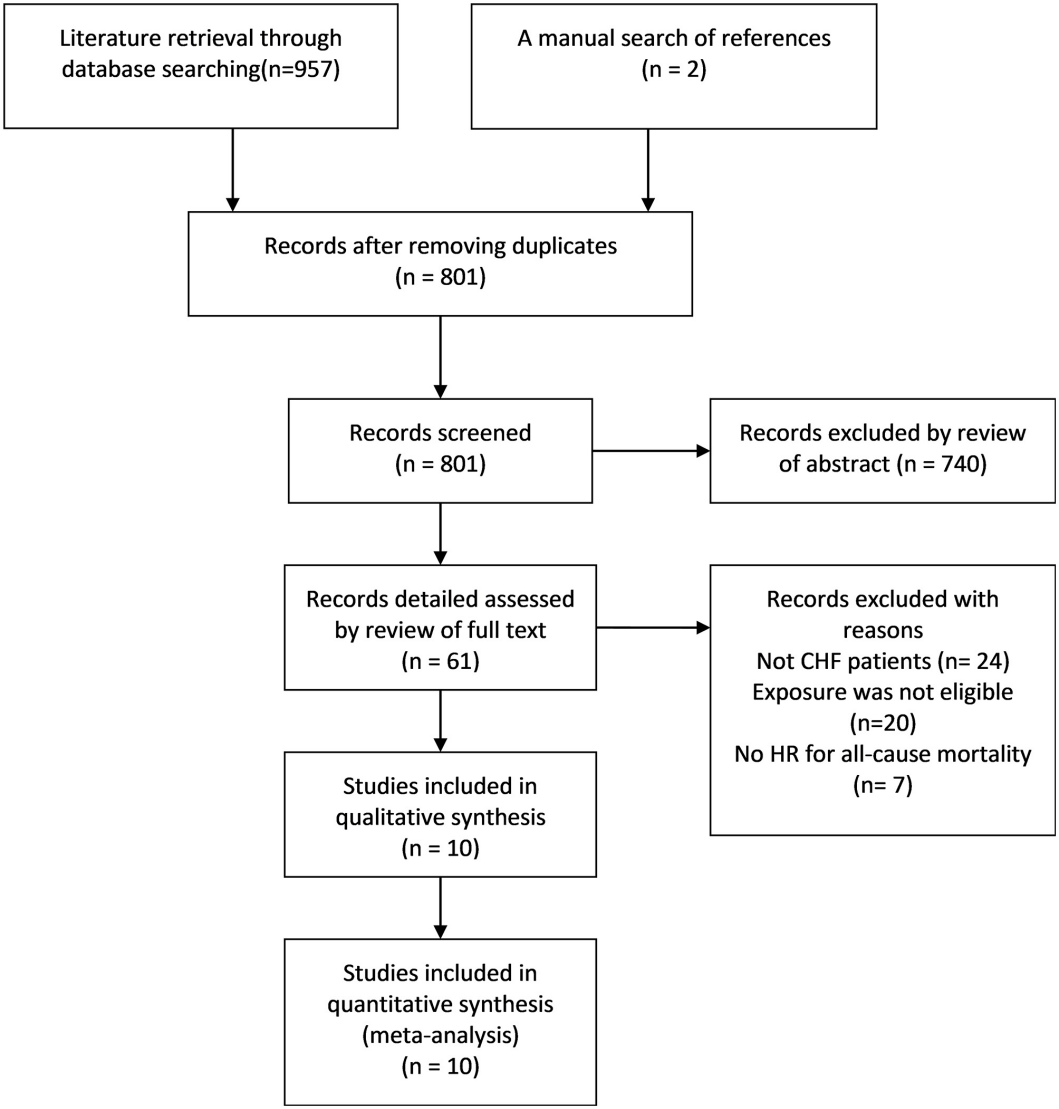
Flow chart of article inclusion and exclusion process.

Researchers initially collected 957 records from the three databases using the predesigned retrieval strategy. After screening all titles and abstracts, 61 articles were selected for detailed assessment. Finally, 10 eligible studies (9, 15–17, 27–32) were included in the qualitative and quantitative analysis. The literature screening process and results are presented in Figure 1.

Of the 10 eligible studies, seven (15–17, 27, 29, 30, 32) were cohort studies and three (9, 28, 31) were *post-hoc* analyses of RCTs, with a total enrollment of 6,244 CHF patients. The median age of the study population ranged from 61 to 75 years, and the percentage of male ranged from 56 to 90.5%. A total of 1,368 deaths occurred in the follow-up period. Detailed information about the 10 studies is listed in Table 1. Table 2 summarizes the results of the methodological quality assessment of each study according to the NOS.

**Table 1:** Baseline characteristics of the study participants.

**First author/year**	**Country**	**Study design**	**Sample size(n)**	**Age (year)**	**Male (%)**	**GDF-15, median(ng/L)**	**LVEF, mean**	**NYHA III or IV (%)**	**Ischemic etiology (%)**	**NT-proBNP median (ng/L)**	**All-Cause Death (n)**	**Follow-up(months)**
Kempf et al. (27)	multicenter	Cohort study	455	64	90.5	1,949	32%	47.70%	67.7	801	117	40
Anand et al. (9)	multicenter	*post-hoc*	1,734	63.2	79	2,040	26%	43%	56	NR	367	23
Lok et al. (28)	Netherlands	*post-hoc*	209	71	73	1,606	<45%	100%	66	1,771	151	100.8
Jungbauer 2014 (15)	Germany	Cohort study	149	61.8	87.2	1,900	<50%	47.60%	61.1	1,504	29	35.1
Chan et al. (29)	Singapore	Cohort study	916	61	76	2,581	<50%	16%	57	NR	81	23
Liu et al. (16)	China	Cohort study	232	65.6	70.3	2,025	33%	41.80%	NR	1,237	53	20
Benes et al. (30)	Czech	Cohort study	121	63.6	85.1	2,094	24.98%	2.88 (mean)	64.5	NR	68	37.4
Bouabdallaoui et al. (31)	multicenter	*post-hoc*	1,938	67	81	sacubitril/valsartan 1,626; enalapril 1,690	30%	25%	63	1,492	315	30
Fernandez et al. (17)	Spain	Cohort study	311	72	56	2,822	58%	39%	25	1,346	98	15
Kuster et al. (32)	France	Cohort study	179	75	69.3	3321	35%	63.8%	53.7	2503	89	80

*LVEF, left ventricular ejection fraction; NYHA, New York Heart Association; NT-pro BNP, N-terminal pro brain natriuretic peptide; NR, not reported*.

**Table 2:** Quality assessment of each included study.

**First author/year**	**Selection**				**Comparability**		**Outcome**			**Stars**
	**Exposed cohort**	**Non-exposed cohort**	**Exposure**	**No outcome was present in the beginning**	**Important confounder adjusted**	**Other confounders adjusted**	**Assessment of outcome**	**Duration of Follow-Up**	**Adequacy of Follow Up**	
Kempf et al. (27)	✰	✰	✰	✰	✰	✰	0	✰	✰	8
Anand et al. (9)	✰	✰	✰	✰	✰	✰	✰	0	0	7
Lok et al. (28)	✰	✰	✰	✰	✰	✰	✰	✰	0	8
Jungbauer et al. (15)	✰	✰	✰	✰	✰	✰	✰	✰	✰	9
Chan et al. (29)	✰	✰	✰	✰	✰	✰	✰	0	0	7
Liu et al. (16)	✰	✰	✰	✰	✰	✰	✰	0	0	7
Benes et al. (30)	✰	✰	✰	✰	✰	✰	✰	✰	0	8
Bouabdallaoui et al. (31)	✰	✰	✰	✰	✰	✰	✰	✰	0	8
Fernandez et al. (17)	✰	✰	✰	✰	✰	✰	✰	0	0	7
Kuster et al. (32)	✰	✰	✰	✰	✰	✰	✰	✰	0	8

### Prognostic Value of GDF-15 in CHF

**Figure 2:**
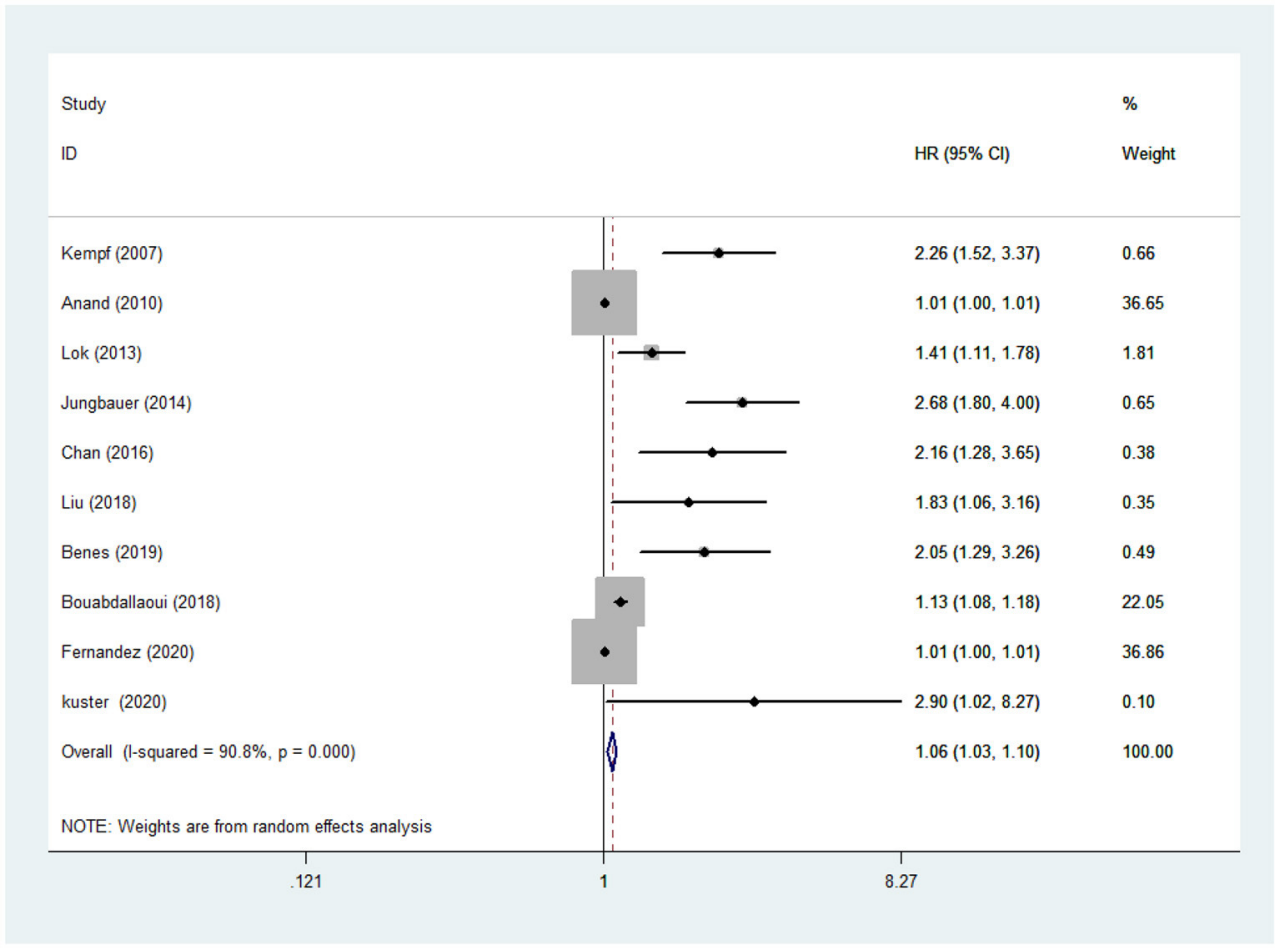
GDF-15 and risk of all-cause death in CHF.

The random-effects models were used to calculate the pooled HR for all-cause mortality because of evident heterogeneity across the studies (*I*^2 =^90.8%, *P* < 0.001). The results in the present study showed that the risk of all-cause mortality in CHF was increased by 6% with a per 1LnU increase in baseline concentration of GDF-15 (HR: 1.06, 95% CI:1.03–1.10, *P* < 0.001) (Figure 2).

### Subgroup Analyses

As summarized in Table 3, there was a stronger association between GDF-15 and risk of death in CHF patients with reduced ejection fraction (HR:1.46, 95%CI: 1.26–1.68, *P* < 0.001) compared to those with non-reduced ejection fraction (HR:1.008, 95%CI: 1.003–1.013, P < 0.05). When we split the NYHA class in low class (NYHA I or II) and high class (NYHA III or IV), no significant heterogeneity was found between the two groups (*P* = 0.56 for interaction). When we stratified the studies by the percentage of CHF patients with ischemic etiology (<60% or ≥60%), we found a close relationship between the elevated concentrations of GDF-15 and the increased all-cause mortality among CHF patients with ischemic etiology (≥60%) (HR:1.75, 95%CI:1.24–2.48, *P* = 0.002). However, this relationship was not shown in CHF patients without ischemic etiology (<60%) (HR:1.01, 95%CI:1.00–1.02, *P* = 0.219). When we stratified the studies by the baseline levels of GDF-15 (< 2000ng/L or ≥ 2000ng/L), the HR for all-cause mortality among patients with lower GDF-15 concentration was 1.69 (1.16–2.47, *P* = 0.007), while the HR among patients with higher GDF-15 was 1.01 (0.99–1.03, *P* = 0.27). When we stratified the studies by the type of study design, the relationship between GDF-15 and all-cause mortality of CHF was detected in the subgroup of cohort study (HR:1.96, 95% CI: 1.25–3.07, *P* = 0.003), whereas that relationship was not identified in the subgroup of *post-hoc* analyses (HR:1.11, 95% CI: 0.99–1,25, *P* = 0.07). In other subgroups stratified by age, publication year, and duration of follow-up, no significant difference was presented.

**Table 3:** Pooled hazard ratios of all-cause mortality in subgroups.

**Subgroups**		**Studies**	**Effects models**	**HR (95% CI) and ***P*** value**	***I*** **^2^**	***P*** **for interaction**
NYHA	NYHA I or II	7 –[9, 15–17,27,29,31]	Random	1.05 (1.02–1.08), *P =* 0.003	92.20%	0.56
	NYHA III or IV	3 [28, 30,32]	Random	1.71 (1.21–2.43), *P =* 0.003	40.90%	
LVEF	<50%	9 [9,15, 16, 27–32]	Random	1.46 (1.26–1.68), *P* < 0.05	91.80%	
	≥50%	Fernandez 2020 [17]		1.008 (1.003–1.013), *P* < 0.05		
Ischemic etiology (%)	<60%	3 [9,17,29]	Random	1.01 (1.00–1.02), *P =* 0.219	75.20%	0.36
	≥60%	6 [15,27–28,30–32]	Random	1.75 (1.24–2.48), *P =* 0.002	89.30%	
GDF**-**15	<2 000	4[15,27–28,31]	Random	1.69 (1.16–2.47), *P =* 0.007	90.50%	0.67
	≥2 000	6 [9,16–17,29–30,32]	Random	1.01 (0.99–1.03), *P =* 0.27	80.50%	
Age	<65	5 [9,15,27,29,30]	Random	1.90 (1.12–3.22), *P =* 0.018	92.90%	0.23
	≥65	5 [16–17,28,31–32]	Random	1.15 (1.02–1.30), *P =* 0.019	90.30%	
Follow-up	≤ 36	6 [9,15–17,29,31]	Random	1.04 (1.01–1.07), *P =* 0.006	91.80%	0.27
	>36	4 [27–28,30,32]	Random	1.86 (1.37–2.53), *P* < 0.001	50.00%	
Study design	Cohort study	7 [15–17,27,29–30,32]	Random	1.96 (1.25–3.07), *P =* 0.003	90.70%	0.07
	*post-hoc*	3 [9,28,31]	Random	1.11 (0.99–1,25), *P =* 0.071	94.00%	
Publication year	≤ 2015	4 [9,15,27–28]	Random	1.66 (1.06–2.59), *P =* 0.027	93.60%	0.84
	>2015	6 [16–17,29–32]	Random	1.21 (1.06–1.39), *P =* 0.005	90.10%	
Sample size	≤ 500	7 [15–17,27–28,30,32]	Random	1.80 (1.25–2.60), *P =* 0.002	90.60%	0.24
	>500	3 [9, 29, 31]	Random	1.10 (0.97–1.25), *P =* 0.12	94.00%	

### Regression Analyses and Sensitivity Analyses

**Figure 3:**
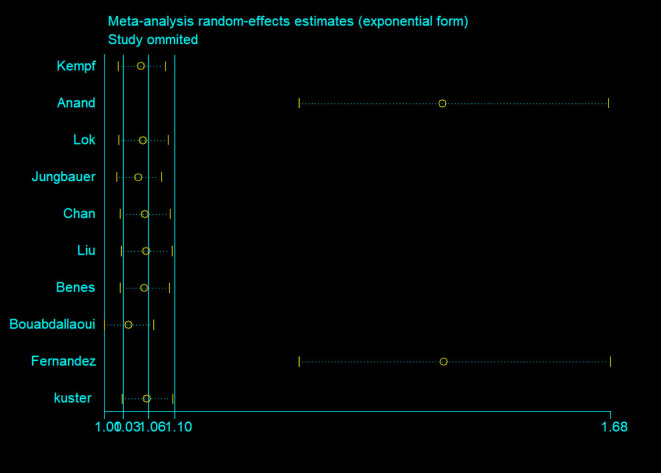
Sensitivity analysis.

The results in the meta-regression analyses did not detect correlations between HR of all-cause mortality with sample size (*P* = 0.107), year of publication (*P* = 0.8), age (*P* = 0.288), follow-up duration (*P* = 0.571), proportion of male (*P* = 0.142), NT-proBNP (*P* = 0.833), and body mass index (BMI) (*P* = 0.301). The sensitivity analysis showed that although Anand et al. (9) and Fernandez et al. (17) had great effects on the pooled HR, the exclusion of each study neither reduced the heterogeneity between studies nor substantially changed the overall estimation of this meta- analysis (Figure 3).

**Figure 4:**
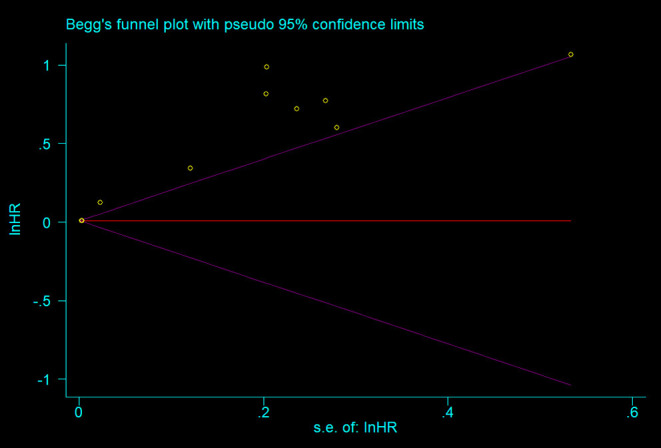
Begg's funnel plot of the meta-analysis.

**Figure 5:**
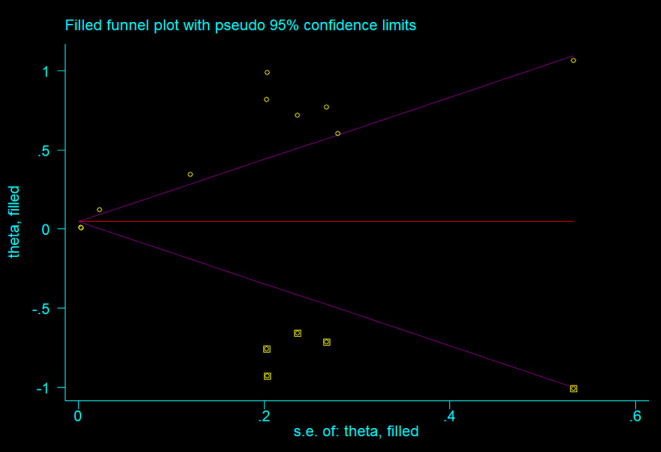
Funnel plot after correction of publication bias.

As shown in the Figure 4, all the points were obviously asymmetrically distributed in the Begg's funnel plot, indicating the publication bias in our meta-analysis which was consistent with the Egger's test (*P* < 0.05). The distribution became symmetrical after correction with five additional points in the Begg's funnel plot (Figure 5), suggesting that at least five more articles were needed. Notably, the correction of publication bias did not substantially alter the conclusion of our meta- analysis (HR after correction: 1.05,95%CI:1.01–1.09, *P* = 0.017), proving the robustness of our assessment.

## Discussion

This meta-analysis indicates that the elevated circulating GDF-15 concentration is associated with the increased risk of all-cause mortality in CHF patients, especially, among those with ischemic etiology (e.g. coronary atherosclerosis). Furthermore, the association becomes stronger after removing the study from Fernandez et al. (17) conducted in patients with non-reduced EF, suggesting that GDF-15 might have a greater prognostic value in individuals with reduced EF. Subgroup analyses show that age, NYHA class, and duration of follow-up may not affect the relationship of GDF-15 with the risk of all-cause death in CHF.

Consistent with earlier studies (15, 16), our findings demonstrate that GDF-15 is a valuable predictive factor of adverse outcomes in stable CHF patients. In the study of Jungbauer et al. (15), there was a 2.68-fold high risk for all-cause death among CHF patients with elevated GDF-15. Similarly, in a study of HF patients with a history of MI (16), there was a nearly 2-fold high risk for all-cause death among CHF patients with elevated GDF-15. In contrast, studies by Anand et al. (9) and Fernandez et al. (17) did not observe a strong association between GDF-15 and mortality in CHF population. Sample size, participants' characteristics and study design might contribute to the inconsistent findings. In line, the subgroup analyses in our study provided an evidence that study design would affect the overall estimation of association between GDF-15 and mortality in CHF. Compared with previous meta-analysis investigating the relationship between GDF-15 and mortality in all HF patients (18), we focused on the individuals with chronic stable HF, which made the population in our study more homogeneous. The up-to-date evidence of 5 new studies (16, 17, 30–32) were included in our meta-analysis. Moreover, subgroup analyses were performed to understand the impact of NYHA class, ischemic etiology and age on the prognostic value of GDF-15.

Ischemic heart disease is one of the major causes of CHF. Myocardial ischemia induced by coronary atherosclerosis or microcirculation dysfunction is tightly associated with heart structure and function. Several observational studies showed that GDF-15 concentration was continuously associated with the risk of coronary heart disease (33, 34). Our data suggest that GDF-15 is a strong predictive factor of the mortality of CHF with ischemic etiology. On the contrary, Anand et al. (9) performed a *post-hoc* analysis of Valsartan Heart Failure Trial (Val-HeFT) and provided an argued evidence that GDF-15 was a biomarker in prediction of all-cause death in CHF with non-ischemic etiology. These conflicting results might be attributed to the study design. To further elucidate the prognostic value of GDF-15 in CHF patients with or without ischemic etiology, chronic ischemic HF population and chronic non-ischemic HF population should be studied, respectively, in future.

Certain studies have reported that the concentrations of GDF-15 increased with age and GDF-15 was an independent predictor of mortality in older individuals without history of CVD (35, 36). However, in the present study, the findings from subgroups showed that the prognostic value of GDF-15 was not affected by age (*P* = 0.23 for interaction), so GDF-15 might be served as an independent risk indicator in CHF patients with different age. There were several evidences that the circulating GDF-15 concentration was positively correlated with the NYHA class among CHF patients (12, 16), suggesting that the NYHA class might influence the predictive power of GDF-15 for HF outcome. Nevertheless, our subgroup analyses did not show heterogeneity in terms of prognostic value of GDF-15 in CHF presented with NYHA class I/II or III/IV (*P* = 0.56 for interaction). It was noteworthy that of the 10 studies, only three were conducted in NYHA class III/IV patients. The predictive role of GDF-15 in CHF with different NYHA functional classification need further assessments.

As for the cut-off value of GDF-15, Liu et al. (16) found the optimal cut-off value of 1,964 ng/L in prediction of all-cause death in CHF. To determine whether GDF-15 concentrations affect the predictive value of GDF-15 for all-cause death in CHF, the population in present study were divided into two groups according to a value of 2,000 ng/L. Unexpectedly, our findings indicated that baseline GDF-15 was closely associated with all-cause death in the subset with median GDF-15 below 2000 ng/L, while the association was not found in the subset with median GDF-15 above 2000 ng/L. The races and prior history of MI might explain the inconsistency between our results and Liu *et al's* data. Most of the studies included in our meta-analysis were done in Europe including Spain, Czech, and Germany, only the study from Liu et al. (16) was conducted only in Chinese Han race patients with post-MI chronic HF. Optimal cut-off value of GDF-15 in the risk stratification of CHF are needed.

Currently, the pathogenic mechanisms underlying the prognostic power of GDF-15 in HF haven't been elucidated. Previous studies in animals have reported contradictory results with respect to the effect of GDF-15 in cardiomyocyte cells (37, 38). Heger et al. (37) reported a stimulating role of GDF-15 on hypertrophic growth of cardiomyocytes *via* the phosphatidylinositol 3-kinase (PI3K), extracellular signal regulated kinase (ERK), and the transcription factor R-SMAD1 (small mother against decapentaplegic, SMAD) signaling pathways; whereas, Xu et al. (38) found an inhibiting effect of GDF-15 on myocardium hypertrophy *via* activating SMAD2/3. The pro-hypertrophic and anti-hypertrophic effects of GDF15 suggested that GDF15 might play a mediating role in myocardial hypertrophy responses to different environmental conditions.

Although the present meta-analysis demonstrates the ability of GDF-15 to predict mortality in stable CHF population, there are still some questions that should be addressed before routine clinical use in this setting. First, optimal cut-off value of GDF-15 for the risk stratification is lacking. Second, although GDF-15 has a more pronounced prognostic significance in CHF patients with ischemic etiology as described in this meta-analysis, the prognostic significance of GDF-15 is not standardly analyzed for ischemic CHF patients and non-ischemic CHF patients separately to increase accuracy. Third, we performed subgroup analyses stratified by age of 65 and found no significant difference between the two groups (<65 or ≥65). However, we did not stratified studies by age of 50 or 75 since the median age of the study population included in this meta-analysis ranged from 61 to 75 years. Thus, larger-scale prospective studies should be conducted to validate the effect of age on the predictive ability of GDF-15. Furthermore, the effect of comorbidities such as chronic kidney diseases and atrial fibrillation on the prognostic value of GDF-15 in CHF should be considered.

### Strengths and Limitations

Firstly, this meta-analysis extensively collected eligible studies conducted in different countries. Secondly, the subgroup analyses provided evidence of GDF-15 as a potential biomarker in CHF with different clinical status. Finally, each individual study included in our analysis had a good quality; the modification for publication bias using the “trim-and-fill” method did not change the overall estimation of HR, proving the robustness of our assessment. Nevertheless, there are several limitations should be noted. Obvious heterogeneity across all the included studies was detected and the meta–regression analyses and subgroup analyses could not distinguish the sources of heterogeneity. Some inherent differences including different assay methods of GDF-15, different medications, and other comorbidities may contribute to the heterogeneity. Thus, one should be cautious to interpret the results in our study. Furthermore, we failed to assess the relationship between GDF-15 and HF rehospitalization due to the limited data.

## Conclusion

This meta-analysis indicates that the elevated level of GDF-15 is associated with the increased risk of all-cause mortality in CHF patients and the predictive power of GDF-15 is more significant among ischemic CHF patients than non-ischemic CHF patients. The circulating GDF-15 could be a predictive factor in CHF patients.

## Data Availability Statement

The original contributions presented in the study are included in the article/Supplementary Material, further inquiries can be directed to the corresponding author/s.

## Author Contributions

J-WL, W-HD, and D-ZS conceived the idea of this research. J-WL developed a protocol with the assistance of D-ZS and W-HD. J-WL and Y-QY performed the data analyses independently. J-WL and LS participated in the data interpretation and drafting of manuscript. D-ZS and W-HD critically reviewed the manuscript. All authors had browsed and approved the final version of the manuscript.

## Funding

This research is supported by the project of TCM and Western Medicine clinical cooperation for major and intractable diseases, angina after coronary revascularization.

## Conflict of Interest

The authors declare that the research was conducted in the absence of any commercial or financial relationships that could be construed as a potential conflict of interest.

## Publisher's Note

All claims expressed in this article are solely those of the authors and do not necessarily represent those of their affiliated organizations, or those of the publisher, the editors and the reviewers. Any product that may be evaluated in this article, or claim that may be made by its manufacturer, is not guaranteed or endorsed by the publisher.

## References

[1] Chinese Society of Cardiology Editorial Board of Chinese Journal of Cardiology. A guide to diagnosis and treatment of heart failure in China 2018. Chin J Cardiol. (2018) 46:760–89.

[2] Yancy CW Jessup M Bozkurt B Butler J Casey DE Jr Colvin MM . 2017 ACC/AHA/HFSA Focused Update of the 2013 ACCF/AHA Guideline for the Management of Heart Failure: A Report of the American College of Cardiology/American Heart Association Task Force on Clinical Practice Guidelines and the Heart Failure Society of America. Circulation. (2017) 136:e137–61. 10.1161/CIR.0000000000000509 28455343

[3] Ponikowski P Voors AA Anker SD Bueno H Cleland JGF Coats AJS . ESC Scientific Document Group. 2016 ESC Guidelines for the diagnosis and treatment of acute and chronic heart failure: The Task Force for the diagnosis and treatment of acute and chronic heart failure of the European Society of Cardiology (ESC)Developed with the special contribution of the Heart Failure Association (HFA) of the ESC. Eur Heart J. (2016) 37:2129–200. 10.1093/eurheartj/ehw128 27206819

[4] Unsicker K Spittau B Krieglstein K. The multiple facets of the TGF-β family cytokine growth/differentiation factor-15/macrophage inhibitory cytokine-1. Cytokine Growth Factor Rev. (2013) 24:373–84. 10.1016/j.cytogfr.2013.05.003 23787157

[5] Wollert KC Kempf T. Growth differentiation factor 15 in heart failure: an update. Curr Heart Fail Rep. (2012) 9:337–45. 10.1007/s11897-012-0113-9 22961192

[6] Frank D Kuhn C Brors B Hanselmann C Lüdde M Katus HA . Gene expression pattern in biomechanically stretched cardiomyocytes: evidence for a stretch-specific gene program. Hypertension. (2008) 51:309–18. 10.1161/HYPERTENSIONAHA.107.098046 18158353

[7] Kempf T Eden M Strelau J Naguib M Willenbockel C Tongers J . The transforming growth factor-beta superfamily member growth-differentiation factor-15 protects the heart from ischemia/reperfusion injury. Circ Res. (2006) 98:351–60. 10.1161/01.RES.0000202805.73038.48 16397141

[8] Adela R Banerjee SK. GDF-15 as a Target and biomarker for diabetes and cardiovascular diseases: a translational prospective. J Diabetes Res. (2015) 2015:490842. 10.1155/2015/490842 26273671 PMC4530250

[9] Anand IS Kempf T Rector TS Tapken H Allhoff T Jantzen F. Serial measurement of growth-differentiation factor-15 in heart failure: relation to disease severity and prognosis in the Valsartan Heart Failure Trial. Circulation. (2010) 122:1387–95. 10.1161/CIRCULATIONAHA.109.928846 20855664

[10] Izumiya Y Hanatani S Kimura Y Takashio S Yamamoto E Kusaka H . Growth differentiation factor-15 is a useful prognostic marker in patients with heart failure with preserved ejection fraction. Can J Cardiol. (2014) 30:338–44. 10.1016/j.cjca.2013.12.010 24484911

[11] Rohatgi A Patel P Das SR Ayers CR Khera A Martinez-Rumayor A . Association of growth differentiation factor-15 with coronary atherosclerosis and mortality in a young, multiethnic population: observations from the Dallas Heart Study. Clin Chem. (2012) 58:172–82. 10.1373/clinchem.2011.171926 22065155 PMC3926660

[12] Wang H Chen Q Li Y Jing X Yang J. Prognostic value of growth differentiation factor-15 in Chinese patients with heart failure: a prospective observational study. Cardiol J. (2018) 25:245–53. 10.5603/CJ.a2017.0068 28612904

[13] Sharma A Stevens SR Lucas J Fiuzat M Adams KF Whellan DJ . Utility of growth differentiation factor-15, a marker of oxidative stress and inflammation, in chronic heart failure: insights from the HF-ACTION study. JACC Heart Fail. (2017) 5:724–34. 10.1016/j.jchf.2017.07.013 28958347 PMC8023398

[14] Du H Yang L Zhang H Zhang XL Shao HY. Correlation between growth differentiation factor-15 and the severity of chronic heart failure in patients with coronary atherosclerosis. Eur Rev Med Pharmacol Sci. (2020) 24:12844–8. 10.26355/eurrev_202012_24186 33378034

[15] Jungbauer CG Riedlinger J Block D Stadler S Birner C Buesing M . Panel of emerging cardiac biomarkers contributes for prognosis rather than diagnosis in chronic heart failure. Biomark Med. (2014) 8:777–89. 10.2217/bmm.14.31 25224934

[16] Liu JX Li YP Liu BH Zhao XJ Zhang ZY Wang JD . Repeated measurement of growth-differentiation factor-15 in Chinese Han patients with post-myocardial infarction chronic heart failure. J Geriatr Cardiol. (2018) 15:618–27. 10.11909/j.issn.1671-5411.2018.10.002 30416510 PMC6221845

[17] Mendez Fernandez AB Ferrero-Gregori A Garcia-Osuna A Mirabet-Perez S Pirla-Buxo MJ Cinca-Cuscullola J . Growth differentiation factor 15 as mortality predictor in heart failure patients with non-reduced ejection fraction. ESC Heart Fail. (2020) 7:2223–9. 10.1002/ehf2.12621 32589369 PMC7524215

[18] Zeng X Li L Wen H Bi Q. Growth-differentiation factor 15 as a predictor of mortality in patients with heart failure: a meta-analysis. J Cardiovasc Med (Hagerstown). (2017) 18:53–9. 10.2459/JCM.0000000000000412 27454651

[19] Liberati A Altman DG Tetzlaff J Mulrow C Gøtzsche PC Ioannidis JP . The PRISMA statement for reporting systematic reviews and meta-analyses of studies that evaluate healthcare interventions: explanation and elaboration. BMJ. (2009) 339:b2700. 10.1136/bmj.b2700 19622552 PMC2714672

[20] Habal MV Garan AR. Long-term management of end-stage heart failure. Best Pract Res Clin Anaesthesiol. (2017) 31:153–66. 10.1016/j.bpa.2017.07.003 29110789 PMC5726453

[21] WRITING COMMITTEE MEMBERS Bozkurt B Coats A Tsutsui HA. Report of the Heart Failure Society of America, Heart Failure Association of the European Society of Cardiology, Japanese Heart Failure Society and Writing Committee of the Universal Definition of Heart Failure Consensus Conference. Eur J Heart Fail. (2021) 23:352–80. 10.1002/ejhf.2168 33605000

[22] Stang A. Critical evaluation of the Newcastle-Ottawa scale for the assessment of the quality of nonrandomized studies in meta-analyses. Eur J Epidemiol. (2010) 25:603–5. 10.1007/s10654-010-9491-z 20652370

[23] Higgins JP Thompson SG Deeks JJ Altman DG. Measuring inconsistency in meta-analyses. BMJ. (2003) 327:557–60. 10.1136/bmj.327.7414.557 12958120 PMC192859

[24] Begg CB Mazumdar M. Operating characteristics of a rank correlation test for publication bias. Biometrics. (1994) 50:1088–101. 10.2307/2533446 7786990

[25] Egger M Davey Smith G Schneider M Minder C. Bias in meta-analysis detected by a simple, graphical test. BMJ. (1997) 315:629–34. 10.1136/bmj.315.7109.629 9310563 PMC2127453

[26] Zheng TS Zhong WZ. Performance of the nonparametric trim and fill method in stata. J Evid Based Med. (2009) 9: 240–242.

[27] Kempf T von Haehling S Peter T Allhoff T Cicoira M Doehner W . Prognostic utility of growth differentiation factor-15 in patients with chronic heart failure. J Am Coll Cardiol. (2007) 50:1054–60. 10.1016/j.jacc.2007.04.091 17825714

[28] Lok DJ Klip IT Lok SI Bruggink-André de la Porte PW Badings E. Incremental prognostic power of novel biomarkers (growth-differentiation factor-15, high-sensitivity C-reactive protein, galectin-3, and high-sensitivity troponin-T) in patients with advanced chronic heart failure. Am J Cardiol. (2013) 112:831–7. 10.1016/j.amjcard.2013.05.013 23820571

[29] Chan MM Santhanakrishnan R Chong JP Chen Z Tai BC Liew OW . Growth differentiation factor 15 in heart failure with preserved vs. reduced ejection fraction. Eur J Heart Fail. (2016) 8:81–8. 10.1002/ejhf.431 26497848

[30] Benes J Kotrc M Wohlfahrt P Conrad MJ Franekova J Jabor A . The Role of GDF-15 in heart failure patients with chronic kidney disease. Can J Cardiol. (2019) 35:462–70. 10.1016/j.cjca.2018.12.027 30935637

[31] Bouabdallaoui N Claggett B Zile MR McMurray JJV O'Meara E Packer M . Growth differentiation factor-15 is not modified by sacubitril/valsartan and is an independent marker of risk in patients with heart failure and reduced ejection fraction: the PARADIGM-HF trial. Eur J Heart Fail. (2018) 20:1701–9. 10.1002/ejhf.1301 30204280

[32] Kuster N Huet F Dupuy AM Akodad M Battistella P Agullo A . Multimarker approach including CRP, sST2 and GDF-15 for prognostic stratification in stable heart failure. ESC Heart Fail. (2020) 7:2230–9. 10.1002/ehf2.12680 32649062 PMC7524044

[33] Li M Duan L Cai YL Li HY Hao BC Chen JQ . Growth differentiation factor-15 is associated with cardiovascular outcomes in patients with coronary artery disease. Cardiovasc Diabetol. (2020) 19:120. 10.1186/s12933-020-01092-7 32746821 PMC7398317

[34] Wang W Song XT Chen YD Yuan F Xu F Zhang M . Growth differentiation factor-15 is a prognostic marker in patients with intermediate coronary artery disease. J Geriatr Cardiol. (2020) 17:210–6. 32362919 10.11909/j.issn.1671-5411.2020.04.004 PMC7189259

[35] Kempf T Horn-Wichmann R Brabant G Peter T Allhoff T Klein G . Circulating concentrations of growth-differentiation factor 15 in apparently healthy elderly individuals and patients with chronic heart failure as assessed by a new immunoradiometric sandwich assay. Clin Chem. (2007) 53:284–91. 10.1373/clinchem.2006.076828 17185363

[36] Wollert KC Kempf T Giannitsis E Bertsch T Braun SL Maier H . An automated assay for growth differentiation factor 15. J Appl Lab Med. (2017) 1:510–21. 10.1373/jalm.2016.022376 33379802

[37] Heger J Schiegnitz E von Waldthausen D Anwar MM Piper HM Euler G. Growth differentiation factor 15 acts anti-apoptotic and pro-hypertrophic in adult cardiomyocytes. J Cell Physiol. (2010) 224:120–6. 10.1002/jcp.22102 20232299

[38] Xu J Kimball TR Lorenz JN Brown DA Bauskin AR Klevitsky R . GDF15/MIC-1 functions as a protective and antihypertrophic factor released from the myocardium in association with SMAD protein activation. Circ Res. (2006) 98:342–50. 10.1161/01.RES.0000202804.84885.d0 16397142

