# Supplementary material for: A Meta-Analysis of Growth Differentiation Factor-15 and Prognosis in Chronic Heart Failure
Source: Front Cardiovasc Med. 2021 Nov 5;8:630818. doi: 10.3389/fcvm.2021.630818 (PMC8602355; doi:10.3389/fcvm.2021.630818)
Supplement: Supplementary file 1 [file Table_1.DOCX]

searching strategy

| Database | Search | Search Strings | Hits(2020.10.1) |
| --- | --- | --- | --- |
| PubMed | #1 | "growth differentiation factor 15"[MeSH Terms] | 1044 |
|  | #2 | (((Macrophage Inhibitory Cytokine 1[Title/Abstract]) OR (Prostate Differentiation Factor[Title/Abstract])) OR (Differentiation Factor, Prostate[Title/Abstract])) OR (GDF-15[Title/Abstract]) | 793 |
|  | #3 | #1 OR #2 | 1309 |
|  | #4 | "heart failure"[MeSH Terms] | 121322 |
|  | #5 | ((((cardiac failure[Title/Abstract]) OR (cardiac dysfunction[Title/Abstract])) OR (congestive heart failure[Title/Abstract])) OR (cardiac insufficiency[Title/Abstract])) OR (left ventricular dysfunction[Title/Abstract]) | 75462 |
|  | #6 | #4 OR #5 | 167887 |
|  | #7 | #3 AND #6 | 105 |
| EMBASE | #1 | 'growth differentiation factor 15'/exp | 3065 |
|  | #2 | 'macrophage inhibitory cytokine 1':ab,ti OR’prostate differentiation factor':ab,ti OR 'differentiation factor, prostate':ab,ti OR 'gdf-15':ti,ab | 1479 |
|  | #3 | #1 OR #2 | 3143 |
|  | #4 | 'heart failure'/exp OR 'cardiac failure':ab,ti OR 'cardiac dysfunction':ab,ti OR 'congestive heart failure':ti,ab OR 'cardiac insufficiency':ti,ab OR 'left ventricular dysfunction':ti,ab | 568320 |
|  | #5 | #3 AND #4 | 625 |
| Cochrane Library | #1 | MeSH descriptor: [Growth Differentiation Factor 15] explode all trees | 42 |
|  | #2 | (Macrophage Inhibitory Cytokine 1) (Word variations have been searched) | 74 |
|  | #3 | (Prostate Differentiation Factor) (Word variations have been searched) | 244 |
|  | #4 | (Differentiation Factor, Prostate) (Word variations have been searched) | 244 |
|  | #5 | (GDF-15) (Word variations have been searched) | 120 |
|  | #6 | MeSH descriptor: [Heart Failure] explode all trees | 9253 |
|  | #7 | (cardiac failure):ti,ab,kw (Word variations have been searched) | 14733 |
|  | #8 | (cardiac dysfunction):ti,ab,kw (Word variations have been searched) | 5539 |
|  | #9 | (cardiac insufficiency):ti,ab,kw (Word variations have been searched) | 2104 |
|  | #10 | (left ventricular dysfunction):ti,ab,kw (Word variations have been searched) | 5032 |
|  | #11 | #1 OR #2 OR #3 OR #4 OR #5 | 251 |
|  | #12 | #6 OR #7 OR #8 OR #9 OR #10 | 26447 |
|  | #13 | #11 AND #12 | 27 |

Second literature retrieval, September 25, 2021

| Pubmed | 35 |
| --- | --- |
| EMBASE | 160 |
| Cochrane Library | 5 |
